# *Bicoid* Signal Extraction with a Selection of Parametric and Nonparametric Signal Processing Techniques

**DOI:** 10.1016/j.gpb.2015.02.006

**Published:** 2015-07-18

**Authors:** Zara Ghodsi, Emmanuel Sirimal Silva, Hossein Hassani

**Affiliations:** 1 The Statistical Research Centre, Bournemouth University, Bournemouth BH8 8EB, UK; 2 Institute for International Energy Studies (IIES), Tehran 1967743 711, Iran

**Keywords:** *Bicoid*, *Drosophila melanogaster*, Signal extraction, Signal processing

## Abstract

The maternal segmentation coordinate gene ***bicoid*** plays a significant role during ***Drosophila*** embryogenesis. The gradient of **Bicoid**, the protein encoded by this gene, determines most aspects of head and thorax development. This paper seeks to explore the applicability of a variety of **signal processing** techniques at extracting ***bicoid*** expression signal, and whether these methods can outperform the current model. We evaluate the use of six different powerful and widely-used models representing both parametric and nonparametric **signal processing** techniques to determine the most efficient method for **signal extraction** in ***bicoid***. The results are evaluated using both real and simulated data. Our findings show that the Singular Spectrum Analysis technique proposed in this paper outperforms the synthesis diffusion degradation model for filtering the noisy protein profile of ***bicoid*** whilst the exponential smoothing technique was found to be the next best alternative followed by the autoregressive integrated moving average.

## Introduction

Morphogens are molecules which determine a cell’s destiny in a concentration-dependent mode by governing the pattern of tissue development and the position of various specialized cell types within a tissue in the process of morphogenesis [1–3]. A classic example of morphogens is bicoid (*bcd*), which is the first known morphogen identified by Nsslein–Volhard in 1988 [1] and encodes a homeobox transcription factor (in what follows, the italic lower-case *bcd* represents either the gene or mRNA and Bcd refers to protein). *bcd* is localised at the anterior end of the egg during the oogenesis [2] and translation of *bcd* begins after fertilization. Consequently, Bcd distributes along the anterior-posterior (AP) axis of the egg, forming a concentration gradient [2]. Such diffusion of Bcd by regulating the production of the anterior structures determines the position and size of head and thorax of an adult fruit fly (http://highered.mcgraw-hill.com).

**Figure 1:**
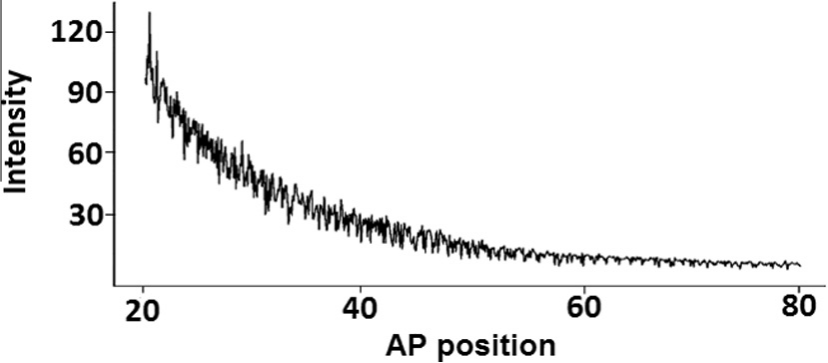
**A typical example of noisy Bicoid** *Y*-axis shows the fluorescence intensities obtained from the attached fluorescence antibodies to the Bcd molecules and *X*-axis shows the position along the embryo.

Several computational models have been published for Bcd gradient over the last three decades (see, for example, [3]). However, as the Bcd profile achieved by fluorescence antibodies technique is highly volatile, some proposed models, such as the simple synthesis diffusion degradation (SDD) model, only exhibited limited performance [4,5]. They fail to clearly explain some characteristics of the Bcd gradient, such as protein life time and length constant [3,6–7]. Figure 1 shows a typical example of the Bcd gradient along the egg length at cleavage cycle 14(3), effect of noise (*i.e.*, fluctuations visible in Figure 1) in this gradient can be seen as the high volatile pattern. An initial look at the distribution suggests Bcd follows an exponential trend. However, owing to the high volatility seen in the series, the extraction of this signal is not a simple task.

SDD, which was formulated before the identification of *bcd* [4,8–11], is the most widely-accepted among the models used to explain Bcd diffusion pattern. SDD ich. As a relatively simple model, SDD follows an exponential curve [12]:

(1) $$B={\mathrm{Ae}}^{-x/\lambda }$$

where A is the amplitude, x is distance from the anterior [13], and λ is the length parameter obtained by fitting an exponential model to the *bcd* intensity profile and computing the position at which the concentration has dropped to 1/exp of the maximal value at the anterior (at x=0) [3]. However, this model is not fully consistent with all the experimental observations. For example based on [4], if Bcd molecules diffuse along the embryo with diffusion constant D and Bcd lifetime of τ, the concentration of Bcd in this model follows:

(2) $$\frac{\partial m(x\text{,}t)}{\partial t}=D\frac{\partial^{2}}{\partial x^{2}}m(x\text{,}t)-\tau_{p}^{-1}m(x\text{,}t)+S(x\text{,}t)$$

where, x and t represent positions along the egg and time, respectively, S(x,t) is a source function describing the production of Bcd molecules, m(x,t) is the formed concentration, and $\tau_{p}$ represents protein lifetime [14]. Nevertheless, when using this model the time needed for attaining the steady state concentration profile is much longer than the protein lifetime τ, whereas the length constant λ is much smaller than the length of the embryo. Moreover, pattern of Bcd expression established by any model should be flexible to different time scales, egg length, and embryos sizes [4,10].

Not only in developmental studies but also in all fields of genetic studies, signal extraction and noise reduction are regarded as important tasks since genetic data are often characterized by the existence of considerable noise. Many methods are utilized for signal extraction, such as machine learning algorithms [15,16] and different background removal techniques [17–19]. In this paper we evaluate the use of powerful and popular signal processing techniques which include both parametric and nonparametric methods to provide a sound extraction of Bcd signal. Our aim is to examine whether the selected signal processing models can provide a more accurate signal extraction of Bcd in comparison to SDD.

The selection of models representing both parametric and nonparametric methods is important for several reasons. Firstly, as seen below, the residuals following signal extraction in Bcd are nonstationary. Secondly, parametric models rely on the underlying assumptions of normality and stationarity, and interestingly, SDD model is parametric. Thirdly, as noted in [20], for the parametric methods, assuming stationarity for the data, linearity of the model and normality of the residuals can provide only an approximation of the true situation. Therefore, a method that does not depend on these assumptions could be very useful for modelling and extracting the signal in Bcd data. Moreover, previous applications in solving signal extraction problems were taken into account when selecting models in this study. We use the SDD model as the overall benchmark as it is the most widely accepted approach for signal extraction in Bcd. We also consider the parametric autoregressive integrated moving average (ARIMA) [21], which has been applied for signal extraction in various fields both historically and recently (see for example, [22–24]). In addition, autoregressive fractionally integrated moving average (ARFIMA), which is mainly recognized as a parametric method suitable for long memory processes where the decay is slower than in an exponential process [24], is included for comparison as well. Other parametric models considered are state space models such as exponential smoothing (ETS), since SDD in itself follows an exponential curve [12]. Singular spectrum analysis (SSA) technique (like neural networks, NN) is a nonparametric signal processing model and does not rely on any assumptions [25]. The SSA technique was initially evaluated for gene expression [26] and has been previously applied for signal extraction [2,6,7,26–30]. Therefore, the models used in this paper include an optimized version of ARIMA [31], an ARFIMA model [31], ETS [32], a feed forward NN model [32], and SSA [33].

Gene expression can be traced either in time or space. The data points used in this study represent the intensity levels for the positions along the AP axis and are considered as a sequenced series. Therefore, one-dimensional gene expression data are used for signal extraction and the second spatial coordinate (Dorsoventral DV axis) has not been considered in this study. Moreover, it is important to note that this paper is not aimed at showing any particular technique to be universally best for modelling the Bcd signal. Instead we are mainly interested in showing how the selected signal processing techniques compare and compete against each other, and the widely accepted SDD model. Any efforts at finding a universally optimal model for this purpose would require more extensive research which considers a wide range of filtering techniques and that objective is beyond the mandate of this paper.

## Methods

### ARIMA

An optimized version of the ARIMA model is provided through the forecast package in R, referred to as auto.arima, and a detailed description of the algorithm can be found in [32]. In brief, the number of differences is defined as *d*, which may be determined using either a Kwiatkowski–Phillips–Schmidt–Shin (KPSS) test, augmented dickey fuller test, or the Phillips–Perron test. The algorithm then minimises the Akaike information criterion (AIC) to determine the values for the order of autoregressive terms p, and the order of the moving average process q. The optimal model is chosen to be the model, which represents the smallest AIC. The decision on the inclusion or exclusion of the constant c is made depending on the value of d.

To expand on the above summary, we provide the following modelling equations for ARIMA based on [33]. A non-seasonal ARIMA model may be written as:

(3) $$(1-\phi_{1}B-\ldots \phi_{p}B^{p}){\left(1-B\right)}^{d}y_{t}=c+(1+\phi_{1}B+\ldots +\phi_{q}B^{q})e_{t}\text{,}$$

or

(4) $$(1-\phi_{1}B-\ldots \phi_{p}B^{p}){\left(1-B\right)}^{d}(y_{t}-\mu t^{d}/d!)=(1+\phi_{1}B+\ldots +\phi_{q}B^{q})e_{t}\text{,}$$

where μ is the mean of ${\left(1-B\right)}^{d}(y_{t}$, $c=\mu (1-\phi_{1}-\ldots -\phi_{p})$ and B is the backshift operator. In the *R* software, the inclusion of a constant in a non-stationary ARIMA model is equivalent to inducing a polynomial trend of order d in the forecast function. It should be noted that when d=0, μ is the mean of $y_{t}$. The seasonal ARIMA model can be expressed as [32]:

(5) $$\Phi (B^{m})\phi (B){\left(1-B^{m}\right)}^{D}{\left(1-B\right)}^{d}y_{t}=c+\Theta (B^{m})\theta (B)\varepsilon_{t}\text{,}$$

where Φ(z) and Θ(z) are the polynomials of orders P and Q, and $\varepsilon_{t}$ is white noise. There is an implied polynomial of order d+D in the forecast function, if c≠0. As mentioned previously, to determine the values of p and q, the AIC of the following form is minimised:

(6) AIC=-2log(L)+2(p+q+P+Q+k),

where k=1 if c≠0 and 0 otherwise, and L represents the maximum likelihood of the fitted model.

### ARFIMA

The general form of an ARFIMA(*p*,*d*,*q*) model shares the same form as an ARIMA process shown in equations (3). However, in contrast to the ARIMA models, here d is allowed to take the form of non-integer values. The ARFIMA model used here is estimated automatically using the Hyndman and Khandakar [31] algorithm explained above, and the Haslett and Raftery [34] algorithm for estimating the parameters including d. Moreover, this Hyndman and Khandakar [31] ARFIMA algorithm combines the functions of fracdiff and auto.arima to automatically select and estimate an ARFIMA model. Initially, the fractional differencing parameter is assumed to be an ARFIMA(2,*d*,0) model. Thereafter the data are fractionally differenced using this estimated d and an ARMA model is selected for the resulting series using auto.arima. Finally, the full ARFIMA(*p*,*d*,*q*) model is re-estimated using the fracdiff function.

### ETS

The ETS technique overcomes a limitation found in earlier ETS models that did not provide a method for easy calculation of prediction intervals [35]. The ETS model from the forecast package considers the error, trend, and seasonal components along with over 30 possible options for choosing the best ETS model via optimization of initial values and parameters using the maximum likelihood estimator and selecting the best model based on the AIC. A detailed description of ETS can be found in [32].

### NN

The NN model has been successfully used for gene expression profiling, clustering and also gene identification [36–38]. NN model is referred to as nnetar and provided through the forecast package for R. A detailed description of the model can be found in [32] along with an explanation on the underlying dynamics. In brief, the nnetar function trains 25 NNs by adopting random starting values and then obtains the mean of the resulting predictions to compute the forecasts. NN takes the form

(7) y^t=β^0+∑j=1kβ^jψ(xtT.γ^j),

where $x_{t}$ consists of p lags of $y_{t}$ and T denotes transpose. Then, the function ψ has the logistic form:

(8) ψ(xt′·γ^j)=1+exp-γ^j0+∑i=1pγ^ji·yt-1-1j=1,…,k.

This form of NN is referred to as one hidden layer feed forward NN model and is the default version in the package. However, we consider the use of multiple hidden layers in order to select the best NN model for these types of data. The nonlinearity arises through the lagged $y_{t}$, entering in a flexible way through the logistic functions of (8). The number of logistic functions (*k*) included is known as the number of hidden nodes.

The NN model in this paper is estimated using the automatic forecasting model, nnetar which is provided through the forecast package in R. For a detailed explanation on how the nnetar model operated, see the ‘Package forecast’ documentation (http://cran.r-project.org/web/packages/forecast/forecast.pdf). The parameters in the NN model are selected based on a loss function embedded into learning algorithm.

### SSA

SSA has been applied for extracting the signal of Bcd and other segmentation genes [2,6,26,27]. The basic SSA method consists of two complementary stages: decomposition and reconstruction; of which each stage includes two separate steps. At the first stage the Bcd is decomposed into the sum of a small number of independent and interpretable components such as a slowly varying trend and a structureless noise [33], and at the second stage the noise-free Bcd is reconstructed [39]. The SSA modelling process for Bcd is summarized below, and in doing so we mainly follow [33].

The first step is concerned with mapping a one dimensional time series $Y_{N}=(y_{1}\text{,}\ldots \text{,}y_{N})$ into the multi-dimensional series $X_{1}\text{,}\ldots \text{,}X_{K}$ with vectors $X_{i}={\left(y_{i}\text{,}\ldots \text{,}y_{i+L-1}\right)}^{T}\in R^{L}$, where K=N-L +1. This process is referred to as embedding whilst the vectors $X_{i}$ are called *L-lagged vectors*. The single choice of the embedding stage is the *window length L*, which is an integer such that 2⩽L⩽N-1. This step results in the trajectory matrix X, which is also a Hankel matrix and takes the form: $X=[X_{1}\text{,}\ldots \text{,}X_{K}]={\left(x_{\mathrm{ij}}\right)}_{i\text{,}j=1}^{L\text{,}K}$.

Next we obtain the singular value decomposition (SVD) of the trajectory matrix and represent it as a sum of rank-one bi-orthogonal elementary matrices. The eigenvalues of ${\mathrm{XX}}^{T}$ are denoted by $\lambda_{1}\text{,}\ldots \text{,}\lambda_{L}$ in decreasing order of magnitude $\left(\lambda_{1}⩾\ldots \lambda_{L}⩾0\right)$ and by $U_{1}\text{,}\ldots \text{,}U_{L}$ the orthonormal system. Set

$$d=\max(i\text{,}\mathrm{suchthat}\;\lambda_{i}>0)=\mathrm{rank}\;X\text{.}$$

If we denote $V_{i}=X^{T}U_{i}/{\sqrt{\lambda }}_{i}$, then the SVD of the trajectory matrix can be written as:

(9) $$X=X_{1}+\cdots +X_{d}\text{,}$$

where $X_{i}={\sqrt{\lambda }}_{i}U_{i}V_{i}^{T}$ (i=1,…,d). The expansion (9) is uniquely defined if all the eigenvalues have a multiplicity of one. The process of splitting the elementary matrices $X_{i}$ into several groups and summing the matrices within each group is called grouping and transfusing each resultant matrix from grouping step to a less noisy series is called diagonal averaging.

Note that usually the first eigenvalue corresponds to the trend of a given dataset when using SSA. Thus we extract the first eigenvalue alone and consider the remainder as noise, and then perform diagonal averaging to transform the matrix containing the first eigenvalue into a time series which will now provide the extracted signal from Bcd and can therefore be compared with the other models.

Details on which models are parametric or nonparametric are presented in Table S1.

## Results

### Simulation results

A series of simulated data are used to evaluate the performance of different techniques. We begin the simulation by considering an exponential curve drawn from the SDD model as the benchmark. In order to obtain a noisy series similar to the real one, random error *ε* of a normal distribution with zero mean and variance $\sigma_{\varepsilon }^{2}$ with different amplitudes were added to different parts of the series [7]. This simulation is repeated 1000 times. Finally, by fitting the different mentioned models to the noisy simulated Bcd series, the following metrics are calculated in order to measure the accuracy of signal extraction. These include the mean absolute error (MAE), mean absolute percentage error (MAPE), and the ratio of the root mean squared error (RRMSE).

(10) MAE=∑i=1M|Yi-Y^i|,

(11) $$\text{MAPE}=\frac{1}{N}\overset{N}{\underset{t=1}{\sum }}\left|100\times \frac{y_{T+h}-y_{T+h\text{,}i}}{y_{T+h}}\right|\text{,}$$

(12) RRMSE=RMSE(AlternateModel)RMSE(SDD)∑i=1N(Sl^-Si)21/2∑i=1N(Sl˜-Si)21/2

where, Sl^ are the estimated values of $s_{i}$, obtained via an alternate model and $\overset{˜}{S_{l}}$ are the estimated values of $s_{i}$ obtained through SDD and N is the series length. The alternate model outperforms the SDD method if RRMSE<1, and performs worse than SDD if RRMSE>1.

Table 1 reports the average RMSE values attained by each model following 1000 iterations and some other descriptives relating to the performance of each model. A significant reduction in the RMSE value is achieved by SSA, confirming that these results are more accurate than those estimated by SDD and other models. Based on the RRMSE criterion, the parametric SDD model reports the worst performance in comparison to the other models considered in this simulation. SDD is outperformed by 66%, 58%, and 52% by the ETS, ARIMA, and ARFIMA, respectively. Interestingly, the nonparametric feed-forward NN model is the second worst performer and outperforms the SDD model by 45%. It should be noted that we have examined the use of multiple hidden layers and selected a NN model with two hidden layers as the most appropriate for these data based on the lowest RMSE and MAE.

According to RRMSE, SSA provides the best signal extraction and is successful at outperforming the SDD model by 80%. The MAE and MAPE criteria also confirm that SSA is the best model in comparison to SDD, ARIMA, ARFIMA, ETS, and NN, whereas SDD is in fact the worst performer in this case. The minimum and maximum columns clearly indicate that there is less variation in the results reported by SSA and accordingly we can confirm that SSA is the most stable model in this case.

In order to verify the statistical significance of the simulation results, we opted for the nonparametric two-sample Wilcoxon test, which could indicate whether the RMSE values attained from two given methods via simulation actually differ in terms of the size. Our results showed a significant difference between the RMSE values obtained via SDD and all other models (*P *= 0.01), further confirming the validity of the results.

The superior performance portrayed by the SSA technique could be explained by several factors. First, SSA model is a specialised filtering technique with the ability of decomposing a given time series and analysing the eigenvalues to accurately identify and separate the noise from the signal. The appropriateness of the separation between signal and noise obtained via SSA was confirmed by the very small values of w-correlation, indicting that the signal and its corresponding noise are almost w-orthogonal [40]. Secondly, it is also interesting to note that the minimum and maximum errors (Table 1) reported by SSA over the 1000 simulations are significantly lower than the minimums and maximums reported by the other models. As a result, it is clear that the SSA technique is more reliable and suitable for signal extraction in Bcd as the average RMSE, MAPE and MAE values are significantly lower than those reported by the other models over the 1000 iterations (more detailed results of the simulation study are available upon request).

### Bicoid data

Next, we used the 17 series (http://urchin.spbcas.ru/flyex/) presented by Alexandrov et al. [26] as the real data for further analysis. A complete explanation on the method and biological characteristics of the 17 series can be found in [30,40,41].

The expression level of Bcd protein in each *Drosophila* embryo was measured by using fluorescently-tagged antibodies. Such quantification relies on the assumption that the actual protein concentrations detected by the antibodies and the fluorescence intensity are linearly related to the Bcd protein concentration in the embryos. In this study nuclear intensities were obtained from a rectangle of 50% of the DV height of the embryo, centred on the AP axis. These data present the gene expression of the AP coordinate between 20% and 80% egg length, which can be considered as a sequenced series. Similar to [26], we set to extract the signal from one-dimensional gene expression data, hence, the second spatial coordinate (DV axis) has not been considered.

**Figure 2:**
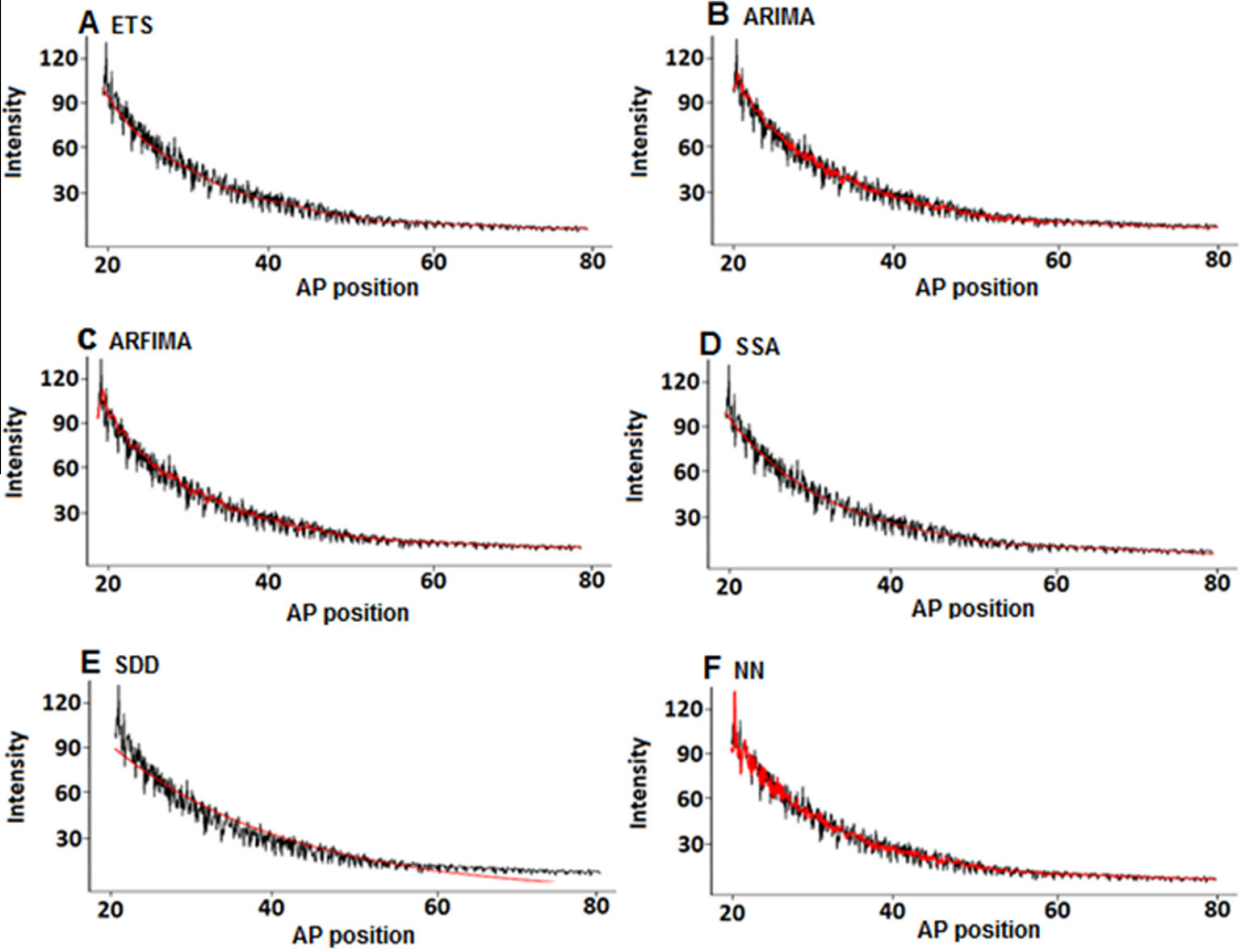
**Signal extraction using various signal processing techniques and SDD model** Black and red colours depict the noisy series and the extracted signal, respectively. SDD, synthesis diffusion degradation.

First we seek to extract the signal in the actual data using various signal processing techniques. The examples of the output from these efforts for embryo hz29 can be found in Figure 2. It is evident that in comparison to the other models, the SSA method provides a relatively smooth signal line. In addition, SDD provides a smooth line as opposed to ARIMA, ARFIMA, and NN models. However, SDD signal appears to be least accurate based on the RMSE, MAE, and MAPE criterions. Overall, the results from the application to real data appear to be consistent with the simulation findings based on data shown in Figure 2.

A close look at Figure 2 suggests that the SDD signal line is the smoothest one out of the evaluated options. However, SDD signal extraction is also the worst fit, as it fails to accurately model the signal amidst the fluctuations, although it appears to have filtered these fluctuations out. The feed-forward NN model with two hidden layers has difficulties in filtering the fluctuations to accurately capture the signal in Bcd. Similar issues exist to various extents for ARIMA and ARFIMA models (ARFIMA being relatively worse than ARIMA). In contrast, ETS and SSA are the most effective ones. Yet, scrutinization of the ETS signal extraction graph revealed that ETS line loses its smoothness to some extent at the middle stages, whereas SSA model is able to provide a smooth signal line right throughout. Therefore, based on the smoothness of the extracted signal, we conclude that SSA does indeed capture the signal in Bcd relatively better than the other methods tested.

**Figure 3:**
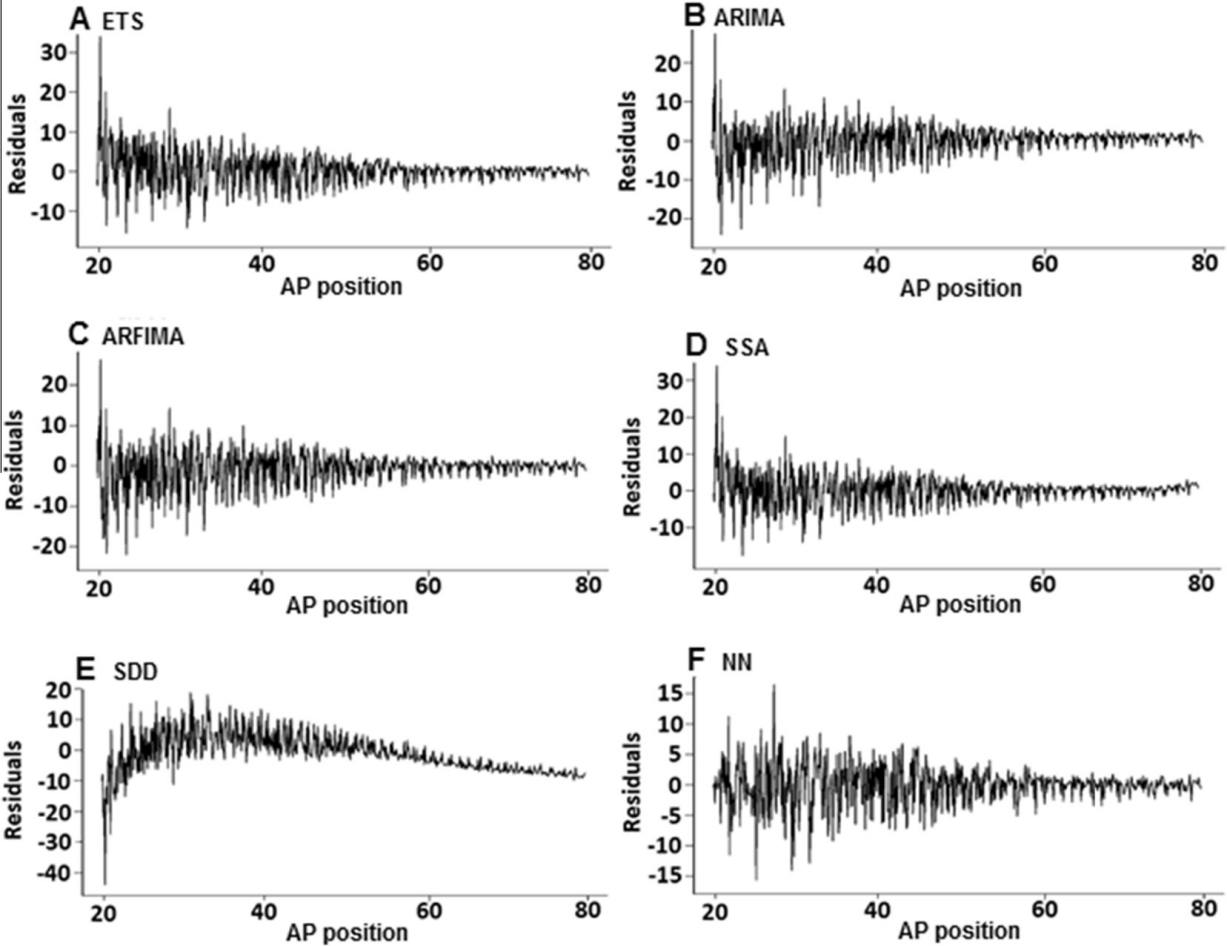
**Residuals following Bicoid signal extraction using various signal processing techniques**.

Figure 3 shows the residuals from each model following signal extraction. The residuals for the Bcd data following signal extraction are nonstationary. To verify this observation, we examined each of the residuals using the augmented Dickey–Fuller (ADF) test for unit roots. Our results showed that the residuals are in fact nonstationary (*P *= 0.01). Interestingly, the parametric models of ARIMA and ARFIMA are able to provide a relatively sound signal extraction for Bcd, although the data are nonstationary. ARIMA algorithm used in this paper automatically considers taking the number of differences until the series becomes stationary, whereas for ARFIMA model, we evaluated a log transformation, which worsened the signal extraction. However, residuals from SDD model do not appear to be white noise but have a clear signal. Accordingly, we tested the residuals from the parametric models for white noise using the Ljung–Box test and indicated that SDD residuals are not white noise (*P *= 0.01). This further explains the comparatively mediocre performance shown by SDD when applied to real data.

Finally, we evaluate the correlation between the signal and noise extracted from each model based on Pearson, Kendall and Spearman correlation coefficients in order to analyze the noise separation capabilities. A correlation coefficient close to zero means that signal and noise do not have a correlation and hence are well seperable. The correlations for all 17 datasets are reported in Table 2. It is evident that all models tested have attained a satisfactory level of separation between noise and signal with SSA providing correlations below 0.10 in 15 out of the 17 cases. Moreover the correlations reported by SSA tend to be smaller (with the exception of a few cases). These results further support the relatively sound performance of SSA in extracting the Bcd signal. The filtering capabilities displayed by SSA are indeed advantageous for signal extraction.

## Conclusion

Even though the extraction of Bcd signal appears to be simple, in practice it is an arduous and complicated task as a result of the nonstationary noise in Bcd. The feasibility of capturing the signal of Bcd gene in *Drosophila* embryos suggests that the SSA model may be of general use in evaluating other expressional systems.

Extracting the Bcd degradation signal from the noisy data is central to this study. We tested various models using both simulated and real data to ensure the validity of the findings. The obtained results illustrate SSA outperforms the SDD model and other methods tested in this study for filtering noisy Bcd. SSA is more flexible than the SDD for the Bcd degradation modelling, whereas ETS was the next best alternative followed by ARIMA. However, it should be noted that we used the simplest form of the SDD model. More advanced models have been developed as the solution to simple SDD model (see for example [42]), which are expected to give more reliable results in Bcd signal extraction.

Moreover, we tested both parametric and non-parametric algorithms in this study, it is of note that non-parametric algorithms are not explicitly better than parametric algorithms. Their performance depends and varies on the nature of the data in question. In the case of Bcd signal extraction, we find the nonparametric SSA model outperforming the rest. Interestingly the simple parametric model of ARIMA outperforms the non-parametric NN algorithm. The poor performance of the NN model can be attributed to its proneness to overfitting.

In conclusion, our results confirm that filtering is very important for Bcd curve fitting and the SSA technique yields a promising result for Bcd analysis. Also, in comparison to other parametric and nonparametric methods evaluated in this paper, using SSA for signal extraction gives the ability of using both dimensions (AP and DV) which leads to a more reliable result. It would be insightful to consider various other filtering approaches, such as nonparametric linear filtering, wavelets, and the NN models (as described in [43,44]) for trend extraction in Bcd in order to examine any differences in performance in comparison not only to ETS and more advanced SDD models, but also to SSA, which has shown promising results.

## Authors’ contributions

HH conceived the idea. ZG completed the task of simulating with SDD and SSA and application to the real data, and ESS handled the fitting of the other time series analysis and forecasting models for both simulation and application to real data. HH wrote the manuscript and all authors participated in the manuscript revision. All authors read and approved the final manuscript.

## Competing interests

The authors declared no competing interests.

## Figures and Tables

**Table 1:** Average loss functions and RRMSE for signal extraction using noisy simulated data

Model	RMSE	MAPE (%)	MAE	RRMSE	Minimum	Maximum
ARIMA	4.69	6.00	3.21	0.42	3.00	7.70
ARFIMA	5.34	5.80	3.42	0.48	3.54	10.19
ETS	3.74	4.40	2.50	0.34	1.89	6.51
NN	6.03	6.27	4.11	0.55	4.02	11.29
SSA	2.25	2.00	1.58	0.20	1.07	3.87
SDD	10.96	23.00	9.30	N/A	10.92	11.27

*Note:* RMSE, root mean squared error; MAPE, mean absolute percentage error; MAE, mean absolute error; RRMSE, ratio of the root mean squared error; ARIMA, autoregressive integrated moving average; ARFIMA, autoregressive fractionally integrated moving average; ETS, exponential smoothing; NN, neural network; SSA, singular spectrum analysis; SDD, synthesis diffusion degradation.

**Table 2:** Correlation values between Bcd signal and noise in 17 different embryos

Embryo	Test	ARIMA	ARFIMA	ETS	NN	SSA	SDD
ac2	Pearson	−0.1026	−0.0716	−0.145	0.047	−0.035	0.057
Kendall	−0.125	−0.087	−0.065	0.001	0.011	0.176
Spearman	−0.146	−0.104	−0.077	0.005	0.03	0.187
ad36	Pearson	−0.225	−0.147	−0.025	0.041	0.051	−0.124
Kendall	−0.161	−0.144	−0.054	0.019	−0.078	−0.452
Spearman	−0.207	−0.193	−0.08	0.022	−0.106	−0.51
as15	Pearson	−0.129	−0.125	−0.125	0.03	0.057	0.161
Kendall	−0.088	−0.086	0.018	0.012	−0.006	0.387
Spearman	−0.114	−0.11	0.032	0.024	0.007	0.438
as18	Pearson	−0.129	−0.125	−0.041	0.03	0.057	0.161
Kendall	−0.088	−0.086	0.018	0.012	−0.006	0.387
Spearman	−0.114	−0.11	0.032	0.024	0.007	0.438
as19	Pearson	−0.157	−0.13	0.015	0.037	0.034	0.065
Kendall	−0.12	−0.099	−0.018	0	−0.054	0.16
Spearman	−0.181	−0.151	−0.024	0.003	−0.079	0.203
as22	Pearson	−0.148	−0.124	−0.069	0.338	−0.03	0.085
Kendall	−0.029	0.008	0.126	0.252	0.093	0.235
Spearman	−0.03	0.018	0.168	0.388	0.14	0.252
as27	Pearson	−0.175	−0.183	−0.053	0.003	0.015	0.195
Kendall	−0.105	−0.073	0.03	0.014	−0.006	0.439
Spearman	−0.139	−0.101	0.042	0.025	0.015	0.473
cb22	Pearson	−0.204	−0.198	−0.025	0.003	0	0.076
Kendall	−0.069	−0.083	0.014	0.011	0.011	0.159
Spearman	−0.086	−0.111	0.031	0.023	0.035	0.163
cb23	Pearson	−0.156	−0.092	−0.041	0.026	0.028	0.06
Kendall	−0.082	−0.037	0.051	0.011	0.039	0.157
Spearman	−0.12	−0.057	0.065	0.01	0.053	0.199
hx8	Pearson	−0.156	−0.092	−0.041	0.026	0.028	0.06
Kendall	−0.082	−0.037	0.051	0.011	0.039	0.157
Spearman	−0.12	−0.057	0.065	0.01	0.053	0.199
hz19	Pearson	−0.186	−0.206	−0.008	0.001	0.038	−0.156
Kendall	−0.092	−0.111	−0.014	0.009	−0.017	−0.335
Spearman	−0.141	−0.168	−0.024	0.007	−0.019	−0.399
hz20	Pearson	−0.152	−0.086	0.063	0.006	−0.005	0.071
Kendall	0.013	0.064	0.177	0.119	0.14	0.123
Spearman	0.02	0.091	0.244	0.162	0.195	0.133
hz29	Pearson	−0.229	−0.188	0.191	−0.001	0.074	0.232
Kendall	−0.062	−0.033	0.116	0.04	0.02	0.463
Spearman	−0.09	−0.054	0.147	0.042	0.025	0.543
iz4	Pearson	−0.202	−0.23	0.022	0.028	−0.003	0.105
Kendall	−0.071	−0.006	0.108	0.034	0.073	0.21
Spearman	−0.086	−0.01	0.16	0.052	0.119	0.212
iz13	Pearson	−0.244	−0.039	−0.026	0.035	0.027	0.149
Kendall	−0.137	−0.008	−0.029	0.008	−0.046	0.334
Spearman	−0.178	0	−0.037	0.024	−0.051	0.387
iz15	Pearson	−0.228	−0.172	0.075	0.029	0.043	0.149
Kendall	−0.119	−0.115	0.049	0.02	−0.006	0.426
Spearman	−0.17	−0.162	0.056	0.026	−0.013	0.523
ms19	Pearson	−0.207	−0.201	0.102	0.028	0.017	0.154
Kendall	−0.141	−0.106	0.069	−0.023	−0.049	0.348
Spearman	−0.185	−0.142	0.096	−0.04	0.048	0.385

*Note:* The signals of 17 Bicoid (Bcd) series in *Drosophila* embryos were presented in [26] and correlation was calculated using R. ARIMA, autoregressive integrated moving average; ARFIMA, autoregressive fractionally integrated moving average; ETS, exponential smoothing; NN, neural network; SSA, singular spectrum analysis; SDD, synthesis diffusion degradation.

## Supplementary material

Table S1 Parametric or non-parametric nature of algorithms.
