# Supplementary material for: Bicoid Signal Extraction with a Selection of Parametric and Nonparametric Signal Processing Techniques
Source: Genomics Proteomics Bioinformatics. 2015 Jul 18;13(3):183–91. doi: 10.1016/j.gpb.2015.02.006 (PMC4563350; doi:10.1016/j.gpb.2015.02.006)
Supplement: Table S1 — Parametric or non-parametric nature of algorithms. [file mmc1.docx]

**Table S1 Parametric or non-parametric nature of algorithms**

| **Model** | **Nature** |
| --- | --- |
| SDD | Parametric |
| ARIMA | Parametric |
| ARFIMA | Parametric |
| ETS | Non-parametric |
| NN | Non-parametric |
| SSA | Non-parametric |

*Note:* SDD, synthesis diffusion degradation; ARIMA, autoregressive integrated moving average; ARFIMA, autoregressive fractionally integrated moving average; ETS, exponential smoothing; NN, neural network; SSA, singular spectrum analysis.
